# Determinants of human milk lactoferrin concentrations in rural Bangladesh

**DOI:** 10.1371/journal.pgph.0005674

**Published:** 2026-01-12

**Authors:** Chloe Andrews, Ingrid Olson, Agata MP Atayde, Salahuddin Ahmed, Nabidul Haque Chowdhury, Rasheda Khanam, Sayedur Rahman, Lars Bode, Mandy B. Belfort, Abdullah H. Baqui, Sarbattama Sen, Anne CC Lee

**Affiliations:** 1 Division of Newborn Medicine, Department of Pediatrics, Brigham & Women’s Hospital, Boston, Massachusetts, United States of America; 2 Harvard T.H. Chan School of Public Health, Boston, Massachusetts, United States of America; 3 Projahnmo Research Foundation, Dhaka, Bangladesh; 4 Johns Hopkins Bloomberg School of Public Health, Baltimore, Maryland, United States of America; 5 Uppsala University, Sweden; 6 Department of Pediatrics, LRF Mother-Milk-Infant Center of Research Excellence (MOMI CORE), Human Milk Institute (HMI), University of California, San Diego, La Jolla, California, United States of America; 7 Harvard Medical School, Boston, Massachusetts, United States of America; PLOS: Public Library of Science, UNITED STATES OF AMERICA

## Abstract

Lactoferrin is an iron-binding glycoprotein in human milk (HM) that reduces the risk of neonatal sepsis. Data from low- and middle-income countries regarding the determinants of HM lactoferrin concentration is limited. Our objectives were to assess how HM lactoferrin concentrations change over time and identify factors associated with lactoferrin concentration. From a pregnancy cohort in Sylhet, Bangladesh, we enrolled 99 women to join the lactation sub-cohort and provide HM samples at a median of 50 (T1) and 146 (T2) days postpartum. We measured HM lactoferrin concentrations with meso-scale discovery and examined associations with predictors including indices of maternal nutritional status [body mass index (BMI), mid-upper arm circumference (MUAC), hemoglobin], depression scores, infant gestational age, and birthweight-for-gestational age z-score. HM lactoferrin concentration increased by 21% from T1 to T2. Higher gestational age at birth was associated with lower HM lactoferrin concentration at T1. Higher maternal MUAC was associated with higher HM lactoferrin concentration at T2. In rural Bangladeshi women, HM lactoferrin concentration increased during the postpartum period. Higher lactoferrin was associated with earlier gestational age at delivery and better maternal nutritional status. Interventions to improve maternal nutritional status might also increase HM lactoferrin concentration and ultimately, benefit child outcomes.

## 1. Introduction

Human milk is the recommended exclusive source of infant nutrition for the first six months of life as it contains macronutrients, micronutrients, and many other bioactive components that support infant growth and development [1]. One of these bioactive components is lactoferrin, an iron-binding protein of the transferrin family with antiviral, antibacterial, and anti-inflammatory properties [2,3]. Lactoferrin makes up 15–20% of the total protein content in human milk [4] and concentrations vary in human milk across individuals and time [5–8].

Bangladesh, a lower-middle-income country [9], has high rates of neonatal sepsis and infectious illness in childhood [10]. Rates and duration of breastfeeding are also high. Among infants 0–5 months of age, 97% are breastfed and 55% are exclusively breastfed [10]. In addition to providing essential nutrients, human milk is the only source of many bioactive components. A better understanding of milk composition is needed to inform breastfeeding-based interventions aimed at better supporting the health of the dyad during the first 1000 days.

Lactoferrin is a potential target in human milk, as it has been shown to reduce late onset sepsis [11], necrotizing enterocolitis [12], respiratory tract illnesses [13,14], and prevalence and severity of diarrhea [13,15], as well as having a beneficial effect on neurodevelopment in infants [16,17]. However, data on the potential determinants of human milk lactoferrin, such as maternal nutritional and mental health status and infant gestational age and birth size, are sparse, mixed, and needed to inform interventions aimed at improving lactoferrin content of human milk and to identify infants at risk of low intake [7,18].

The objectives of our exploratory secondary analysis were to determine how human milk lactoferrin concentrations change over the course of lactation and identify maternal and infant determinants associated with human milk lactoferrin concentrations.

## 2. Materials and methods

### 2.1. Study design and participants

This was a secondary analysis of data from a longitudinal cohort study of 99 mother-infant dyads from the Projahnmo lactation cohort, located in two sub-districts of the rural Sylhet district in northeast Bangladesh (Kanaighat and Zakiganj). The Projahnmo lactation cohort was nested within a larger pregnancy cohort, the Alliance for Maternal and Newborn Health Improvement (AMANHI) Study, for which women were enrolled between 8 and 19 weeks gestation (confirmed by ultrasound) if they intended to stay in the study area for the duration of follow up and consented to collection of data and biological samples [19]. Women were enrolled in the lactation sub-cohort if they delivered a singleton liveborn infant, were over the age of 15 years, were breastfeeding, and had no painful breast conditions, such as mastitis or cracked nipples. The study participants were enrolled into the parent AMANHI study pregnancy cohort from May 17, 2017 to August 30, 2017 and into the subsequent Projahnmo lactation cohort from March 3, 2018 to May 28, 2018.

### 2.2. Ethics statement

The AMANHI study was approved by the Ethical Review Committee of the icddr,b (Protocol PR-12073). The Projahnmo lactation cohort study was approved by the ethics review committee of the Bangladesh Medical Research Council (Protocol # 04907052017) and the institutional review board at Brigham & Women’s Hospital (Protocol # 2014P001741). Written informed consent was obtained from pregnant women in the local language for screening ultrasounds at the study center and for enrollment if they met the eligibility criteria. Community Health Workers (CHWs) read and explained the consent form to participants and addressed any questions they had. The consent also covered follow-up visits and the collection of biological samples. It further specified that all data and specimens would be shared anonymously to protect participants’ privacy and confidentiality, and that participants had the right to withdraw consent at any time.

### 2.3. Data collection

Trained community health workers collected detailed data regarding maternal demographics, dietary habits, and obstetrics history and performed anthropometric assessments on the mothers and infants.

Maternal height, weight, and mid-upper arm circumference (MUAC) were measured at enrollment in the AMANHI Study. MUAC was measured using a TALC insertion tape (precision: 1 mm) and low MUAC was defined as MUAC <23 cm [20,21]. Maternal weight was also measured at 0–6 days and 42–60 days postpartum. Maternal body mass index (BMI) was calculated as weight (kg) divided by height (m) squared at all three timepoints (enrollment, 0–6 days postpartum, and 42–60 days postpartum). BMI categories were determined using the WHO cut-offs for Asian populations (underweight: < 18.5; normal weight: 18.5 to <23; overweight/obese: ≥ 23 kg/m^2^) [22]. Maternal blood samples were collected at two months postpartum and hemoglobin was quantified using the HemoCue analyzer (HemoCue, Ängelholm, Sweden). Anemia was defined as hemoglobin <12 g/dL [23,24]. Mothers completed depression screening with the Patient Health Questionnaire-9 (PHQ-9) at 0–6 days postpartum. We categorized scores as minimal (0–4), mild (5–9), moderate (10–14), and moderately severe or severe (15–27) depression [25]. Mothers reported whether they were providing their infant with anything other than breastmilk at both timepoints from which we were able to ascertain exclusive breastfeeding status. They also reported the date of collection of their human milk samples. We categorized these dates into season of collection based off of the six Bangladesh seasons [26].

Infant gestational age was based upon a dating ultrasound performed at <20 weeks’ gestation and preterm birth was defined as a gestational age < 37 weeks [19]. At delivery, infant birth length was measured and weight was measured with the TANITA BD-585 scale. Infant birthweight-for-gestational age percentiles and z-scores (WAZ) were calculated using the Intergrowth-21^st^ standards. Infants were classified as small-for-gestational age (SGA) if their sex-specific birthweight-for-gestational age percentile was < 10% using the Intergrowth 21^st^ standard [27].

### 2.4. Human milk samples

Mothers provided human milk samples at a median of 50 days postnatal age (IQR 46–55) (T1) and a median of 146 days (IQR 126–168) (T2). During a mid-day feed, mothers were asked to breastfeed their infants for three to five minutes, briefly detach the infant from the breast, and hand express 15 mL of human milk into a sterile container. The samples were split into two falcon tubes of equal volume, flash frozen at -80°C on liquid nitrogen, and shipped to Brigham & Women’s Hospital where they were stored at -80°C until time of analysis.

As previously described [28], we measured lactoferrin concentrations of the human milk samples in duplicate by electrochemiluminescence multiplex immunoassay technology using the mesoscale discovery platform lacto ferrin U-plex assay (Bode Lab, UC San Diego). The capture antibody and the detection antibody are both monoclonal mouse anti-human antibodies.

Results are given as mg/dL of whole milk as validation of the assay showed that dilution of whole human milk yields data parallelism, indicating that other components of human milk (e.g., lipids) do not interfere with the assay. Using whole milk is also advantageous as the process of delipidating milk can introduce variability. Thus, we expect that lactoferrin concentrations in whole milk will be lower than previously reported in skim milk. Due to the high sensitivity of the assay, human milk samples were diluted 1:1,000,000 and a robotic liquid handling system (Assist Plus/Voyager, Integra Biosciences, Hudson, NH, USA) was used to ensure consistent pipetting and avoid errors associated with manual pipetting at such high dilutions (CV < 5%) [28].

We measured the protein content of the human milk samples using the Miris Human Milk Analyzer (Miris AB, Uppsala, Sweden). True protein represents the content of actual protein based only on the amount of protein nitrogen, and excluding non-protein nitrogen, in the sample. The Miris Human Milk Analyzer presents the true protein value by estimating 20% non-protein nitrogen [29].

### 2.5. Statistical analysis

All analyses were performed using Stata 17.0. We assessed data distributions using the Shapiro-Wilks test for normality. We reported sociodemographic and clinical characteristics of the cohort using medians and interquartile ranges (IQRs) for continuous variables and counts and percentages for categorical variables for the entire cohort and by lactoferrin tertile at each timepoint. We assessed differences in characteristic variables across lactoferrin tertiles using the Kruskal-Wallis test for continuous variables and Pearson’s Chi^2^ test for categorical variables.

To determine differences in lactoferrin concentrations over time, we used the Wilcoxon signed-rank test. We performed univariate and multivariable linear regression to determine associations between determinant variables, including maternal BMI, MUAC, hemoglobin, depression scores, and infant gestational age and WAZ, and lactoferrin concentrations at both timepoints. We utilized three models for each linear or logistic regression: model 0 was unadjusted; model 1 was adjusted for important maternal covariates that are known to be associated with the maternal predictors of human milk composition, including age, parity, wealth index, and education; and model 3 was adjusted for the same covariates as in model 1 with the addition of human milk true protein content. Determinant variables were assessed as continuous and categorical variables. We reported β coefficients and 95% confidence intervals (CIs) for all regression models. For all analyses, the significance threshold was designated a priori as p < 0.05. For linear regression models, we assessed regression diagnostics, including residual plots, normality tests, multicollinearity tests, and influence plots.

In a post-hoc power calculation, we determined that we achieved 97% power to determine differences across timepoints given an alpha = 0.05, total sample size of 82, and an effect size of 0.41 for the Wilcoxon signed-rank test.

## 3. Results

### 3.1. Cohort characteristics

Of the 99 mothers enrolled, 95 had at least one lactoferrin measurement available and were included in this analysis (T1: n = 94; T2: n = 83). Maternal and infant characteristics are presented in **Table 1**. Median maternal age was 24 years and the number of years of education was eight. Median maternal BMI at pregnancy enrollment was 19.4 kg/m^2^ and 33% of women were underweight, 60% were normal weight, and 7% were overweight or obese. Twenty-three percent of women had low MUAC at pregnancy enrollment and 72% were anemic at T1. Median postpartum maternal PHQ-9 score was 9. At T1 and T2, 98% and 91% of women were exclusively breastfeeding, respectively. The T1 samples were collected during the Spring and Summer season. There was no difference in season of collection across T1 lactoferrin tertiles. All of the T2 samples were collected during the Monsoon season. Median gestational age at delivery was 39.6 weeks. Approximately 6% of infants were born preterm and 50% were born SGA. Maternal age, parity, BMI category at 0–6 days postpartum, infant birthweight, and SGA status varied significantly across tertiles of T1 lactoferrin (**Table 1**). Maternal age differed significantly across tertiles of T2 lactoferrin (**S1 Table**).

**Table 1:** Population characteristics of mother-infant dyads by tertile of human milk lactoferrin concentration at two months.

	Entire Cohort *n=95*	T1 Lactoferrin Tertile			p-value^a^
		Low	Middle	High	
		*n=32*	*n=31*	*n=31*	
Maternal Characteristics					
Age (years)	24 (20-27)	26 (22.5-30)	22 (20-25)	24 (22-27)	0.014*
Parity					0.001*
Nulliparous	3 (3.2)	0 (0.0)	2 (6.5)	1 (3.2)	
1-2	46 (48.4)	14 (43.8)	8 (25.8)	23 (74.2)	
3 or greater	46 (48.4)	18 (56.2)	21 (67.7)	7 (22.6)	
Education (# years)	8 (5-9)	6 (4.5-8.5)	8 (5-10)	8 (5-9)	0.186
Wealth index	0.1 (-0.6-0.9)	-0.01 (-0.7-0.5)	0.2 (-1.1-1.5)	0.2 (-03-1.0)	0.795
Continuous BMI (kg/m2)					
Baseline	19.4 (17.9-21.8)	19.9 (18.5-22.7)	19.4 (17.9-20.7)	19.1 (17.6-21.9)	0.316
1 month postpartum	21.2 (19.8-22.9)	21.4 (19.9-23.9)	20.8 (19.9-22.4)	21.7 (18.3-23.6)	0.292
4 months postpartum	20.6 (18.6-22.4)	20.6 (18.7-22.9)	20.4 (18.7-21.2)	20.9 (17.6-22.5)	0.632
BMI category (baseline)					0.281
Underweight (<18.5)	31 (32.6)	8 (25.0)	11 (35.5)	12 (38.7)	
Normal weight (18.5-22.9)	57 (60.0)	20 (62.5)	20 (64.5)	17 (54.8)	
Overweight/Obese (≥23)	7 (7.4)	4 (12.5)	0 (0.0)	2 (6.5)	
BMI category (0-6 days postpartum)					0.023*
Underweight (<18.5)	13 (14.4)	2 (6.2)	4 (13.8)	7 (25.0)	
Normal weight (18.5-22.9)	56 (62.2)	20 (62.5)	23 (79.3)	13 (46.4)	
Overweight/Obese (≥23)	21 (23.3)	10 (31.2)	2 (6.9)	8 (28.6)	
BMI category (42-60 days postpartum)					0.114
Underweight (<18.5)	19 (20.0)	4 (12.5)	5 (16.1)	10 (32.3)	
Normal weight (18.5-22.9)	61 (64.2)	21 (65.6)	24 (77.4)	16 (51.6)	
Overweight/Obese (≥23)	15 (15.8)	7 (21.9)	2 (6.5)	5 (16.1)	
MUAC at enrollment (cm)	22.6 (21.5-24.2)	22.4 (21.4-24.2)	22.6 (21.5-23.3)	23.0 (21.5-25.6)	0.557
Low MUAC (<23 cm)	52 (54.7)	18 (56.2)	20 (64.5)	14 (45.2)	0.306
Anemia (Hgb <12 mg/dL)	66 (71.7)	21 (70.0)	20 (64.5)	25 (83.3)	0.240
Depression score	9 (7-12)	8.5 (6-11)	10 (8-12)	9 (6.5-12)	0.589
Depression score category					0.452
Minimal	9 (9.9)	5 (15.6)	3 (10.0)	1 (3.6)	
Mild	38 (41.8)	13 (40.6)	10 (33.3)	15 (53.6)	
Moderate	35 (38.5)	10 (31.3)	15 (50.0)	19 (32.1)	
Moderately severe/severe	9 (9.9)	4 (12.5)	2 (6.7)	3 (10.7)	
Exclusive breastmilk feeding					
1 month postpartum	83 (87.4)	29 (90.6)	26 (83.9)	27 (87.1)	0.691
4 months postpartum (n=85)	33 (38.8)	13 (43.3)	9 (33.3)	10 (37.0)	0.733
Season of T1 milk collection					0.728
Spring (Feb 15-Apr 14)	69 (72.6)	22 (68.8)	24 (77.4)	22 (71.0)	
Summer (Apr 15-Jun 14)	26 (27.4)	10 (31.2)	7 (22.6)	9 (29.0)	
Season of T2 milk collection					
Monsoon (Jun 15-Aug 14)	85 (100.0)	-	-	-	-
Infant Characteristics					
Male sex	48 (50.5)	16 (50.0)	11 (35.5)	20 (64.5)	0.073
GA at delivery (weeks)	39.6 (38.8-40.4)	39.8 (38.9-40.7)	39.3 (38.3-40.4)	39.6 (38.6-40.4)	0.245
Preterm delivery	6 (6.3)	0 (0.0)	3 (9.7)	3 (9.7)	0.185
Birthweight (g)	2720 (2440-2980)	2725 (2565-2885)	2550 (2240-2860)	2820 (2440-3070)	0.023*
SGA	48 (50.5)	19 (59.4)	20 (64.5)	9 (29.0)	0.010*

Data presented are median (IQR) or n (%).

^a^Kruskal Wallis test for continuous variables and Pearson’s Chi^2^ or Fisher’s exact (when a cell had n<5) test for categorical variables.

*p<0.05.

Abbreviations: BMI=body mass index; MUAC=mid upper arm circumference; Hgb=hemoglobin; PHQ-9=Patient Health Questionnaire-9; GA=gestational age; SGA=small for gestational age; IQR=interquartile range.

### 3.2. Human milk lactoferrin concentrations over time

**Fig 1:**
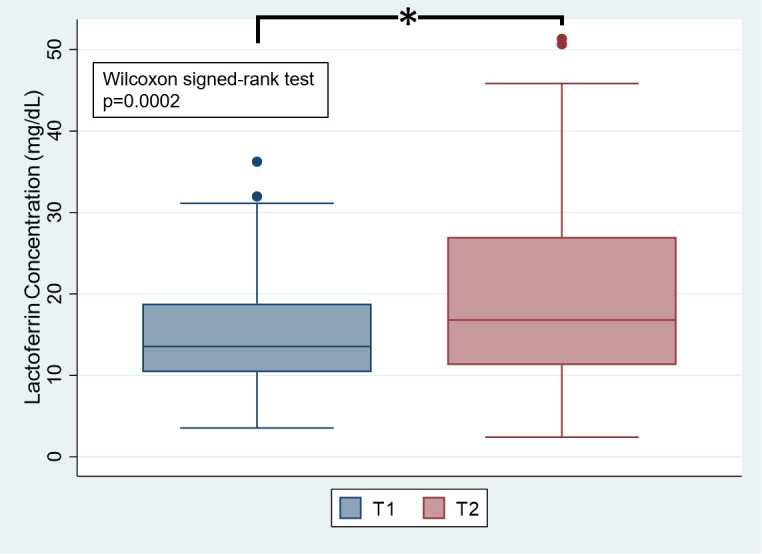
Human milk lactoferrin concentrations at T1 and T2. Legend: * Wilcoxon signed-rank test p < 0.05.

Human milk lactoferrin concentrations were significantly higher at T2 compared to T1 (**Fig 1).** Median lactoferrin concentration was 13.56 (IQR 10.41-18.80) mg/dL at T1 and 16.79 (IQR 11.27-27.00) mg/dL at T2.

### 3.3. Predictors of human milk lactoferrin concentration

In fully adjusted models, higher gestational age at birth was associated with lower human milk lactoferrin concentration at T1 (β -1.01 mg/dL per week increase in GA; 95% CI -1.82, -0.19). Maternal BMI, MUAC, hemoglobin, and depression score, as well as infant WAZ were not associated with human milk lactoferrin at T1 (**Table 2**). Higher MUAC at enrollment was associated with higher human milk lactoferrin concentration at T2 (β 1.22 mg/mL; 95% CI 0.17, 2.27). There was a trend towards higher maternal BMI at enrollment being associated with higher human milk lactoferrin (β 7.70 mg/dL; 95% CI -0.062, 1.60) at T2. Maternal postpartum hemoglobin and depression score, as well as infant gestational age and WAZ were not associated with human milk lactoferrin at T2 (**Table 2**). Of note, maternal MUAC, BMI at baseline, and BMI at both postpartum timepoints were all positively and highly correlated (ρ > 0.7; p < 0.001 for all). Similarly, infant WAZ was positively correlated with maternal MUAC (r = 0.28; p < 0.01), BMI at T1 (r = 0.29; p < 0.01), and BMI at T2 (r = 0.26; p = 0.02).

**Table 2:** Associations of continuous maternal-infant characteristics with human milk lactoferrin concentrations.

	Model 0 β (95% CI)	Model 1 β (95% CI)	Model 2 β (95% CI)
**T1 Lactoferrin (mg/dL) (n = 94)**			
MUAC (cm)	0.37 (-0.20, 0.95)	0.32 (-0.28, 0.92)	0.33 (-0.26, 0.92)
BMI at enrollment (kg/m^2^)	0.026 (-0.46, 0.52)	0.0067 (-0.51, 0.50)	-0.018 (-0.52, 0.48)
BMI at 0–6 days (kg/m^2^)	-0.069 (-0.57, 0.44)	-0.14 (-0.66, 0.38)	-0.14 (-0.65, 0.37)
Hemoglobin at 42–60 days (g/dL)	-0.55 (-1.65, 0.55)	-0.47 (-1.52, 0.57)	-0.58 (-1.61, 0.45)
Depression score	0.11 (-0.23, 0.45)	0.03 (-0.30, 0.37)	-0.012 (-0.35, 0.32)
GA at delivery (weeks)	-0.97 (-1.88, -0.069)*	-0.96 (-1.85, -0.061)*	**-1.01 (-1.82, -0.19)** ^**a**^ *
BW for GA (z-score)	1.15 (-0.21, 2.50)^	0.62 (-0.74, 1.98)	0.69 (-0.65, 2.03)
**T2 Lactoferrin (mg/dL) (n = 83)**			
MUAC (cm)	0.90 (-0.13, 1.94)^	1.21 (0.17, 2.26)*	1.22 (0.17, 2.27)*
BMI at enrollment (kg/m^2^)	0.47 (-0.36, 1.30)	0.72 (-0.11, 1.55)^	0.77 (-0.062, 1.60)^
BMI at 42–60 days (kg/m^2^)	0.49 (-0.34, 1.32)	0.61 (-0.24, 1.46)	0.64 (-0.21, 1.49)
Hemoglobin at 42–60 days (g/dL)	-0.10 (-2.09, 1.89)	0.03 (-1.94, 2.00)	0.08 (-1.90, 2.07)
Depression score	0.21 (-0.42, 0.83)	0.21 (-0.44, 0.86)	0.10 (-0.56, 0.75)
GA at delivery (weeks)	-1.06 (-2.75, 0.63)	-0.84 (-2.53, 0.85)	-0.93 (-2.63, 0.76)
BW for GA (z-score)	0.60 (-1.98, 3.19)	0.41 (-2.17, 2.99)	0.26 (-2.35, 2.88)

Model 0 = unadjusted; Model 1 = adjusted for maternal age, parity, wealth index, and education; Model 3 = model 2 + breastmilk true protein at each timepoint.

Abbreviations: MUAC = mid upper arm circumference; BMI = body mass index; GA = gestational age; BW = birthweight. * = p < 0.05; ^ = p < 0.10.

^a^Due to the presence of heteroskedasticity, robust standard errors were used.

When examining the predictor variables as categorical variables, there were no significant associations between the predictors and human milk lactoferrin concentration at either timepoint in fully adjusted models (**Table 3**). In unadjusted models only, milk from mothers of infants who were born SGA had significantly lower human milk lactoferrin concentrations at T1 (β -3.26 mg/dL; 95% CI -5.89, -0.63) compared to mothers of infants who were not born SGA. In fully adjusted models, there was a trend towards preterm human milk having higher lactoferrin concentration at T1 compared to term human milk (β 4.40 mg/dL; 95% CI -0.86, 9.67; p = 0.10). Lastly, there was a trend towards mothers who were overweight or obese at 42–60 days postpartum having higher human milk lactoferrin at T2 compared to mothers of normal weight (β 6.07 mg/dL; 95% CI -0.76, 12.91; p < 0.10).

**Table 3:** Associations of categorical maternal-infant characteristics with human milk lactoferrin concentrations.

	Model 0 β (95% CI)	Model 1 β (95% CI)	Model 2 β (95% CI)
**T1 Lactoferrin (mg/dL)**			
MUAC			
Low (<23 cm)	-2,30 (-4.99, 0.38)^	-1.50 (-4.42, 1.41)	-0.98 (-3.90, 1.95)
Normal (≥23 cm)	Ref	Ref	Ref
BMI category at enrollment			
Underweight (<18.5 kg/m^2^)	0.53 (-2.41, 3.47)	0.75 (-2.11, 3.61)	0.90 (-1.92, 3.73)
Normal weight (18.5-22.9 kg/m^2^) Overweight/Obese (≥23 kg/m^2^)	Ref -1.75 (-7.40, 3.91)	Ref -2.24 (-7.68, 3.21)	Ref -1.25 (-6.71, 4.21)
BMI category at 0–6 days			
Underweight	2.55 (-1.47, 6.57)	2.77 (-1.22, 6.76)	2.53 (-1.41, 6.48)
Normal weight	Ref	Ref	Ref
Overweight/Obese	0.92 (-2.49, 4.32)	-0.15 (-3.67, 3.38)	-0.017 (-3.52, 3.49)
Anemia at 42–60 days			
Anemic (<12 mg/dL)	2.38 (-0.63, 5.39)	1.68 (-1.24, 4.60)	2.03 (-0.86, 4.91)
Not anemic (≥12 mg/dL)	Ref	Ref	Ref
Depression score category			
Minimal/mild	Ref	Ref	Ref
Moderate/severe	-0.22 (-2.97, 2.53)	-1.01 (-3.80, 1.78)	-1.27 (-4.01, 1.47)
GA at delivery			
Preterm (<37 weeks)	3.40 (-2.10, 8.90)	3.67 (-1.68, 9.02)	4.41 (-0.86, 9.67)
Term (≥37 weeks)	Ref	Ref	Ref
BW for GA			
SGA (<10^th^ percentile)	-3.26 (-5.89, -0.63)*	-1.94 (-4.69, 0.81)	-1.92 (-4.63, 0.79)
Not SGA (≥10^th^ percentile)	Ref	Ref	Ref
**T2 Lactoferrin (mg/dL)**			
MUAC			
Low (<23 cm)	-0.59 (-5.58, 4.40)	-2.25 (-7.66, 3.16)	-2.75 (-8.21, 2.71)
Normal (≥23 cm)	Ref	Ref	Ref
BMI category at baseline			
Underweight (<18.5 kg/m^2^)	-2.27 (-7.78, 3.05)	-3.16 (-8.45, 2.13)	-3.41 (-8.72, 1.90)
Normal weight (18.5-22.9 kg/m^2^)	Ref	Ref	Ref
Overweight/Obese (≥23 kg/m^2^)	2.86 (-6.26, 11.99)	3.77 (-5.10, 12.64)	3.99 (-4.91, 12.88)
BMI category at 42–60 days			
Underweight	2.78 (-3.76, 9.31)	2.46 (-4.05, 8.98)	1.93 (-4.73, 8.59)
Normal weight	Ref	Ref	Ref
Overweight/Obese	5.43 (-1.29, 12.14)	6.58 (-0.10, 13.26)^	6.07 (-0.76, 12.91)^
Anemia at 42–60 days			
Anemic (<12 mg/dL)	-0.93 (-6.44, 4.58)	-2.21 (-7.66, 3.25)	-1.82 (-7.35, 3.71)
Not anemic (≥12 mg/dL)	Ref	Ref	Ref
Depression score category			
Minimal/mild	Ref	Ref	Ref
Moderate/severe	-0.30 (-5.34, 4.75)	-2.23 (-7.49, 3.04)	-2.40 (-7.75, 2.96)
GA at delivery			
Preterm (<37 weeks)	4.84 (-6.72, 16.39)	4.65 (-6.81, 16.11)	5.45 (-6.08, 16.98)
Term (≥37 weeks)	Ref	Ref	Ref
BW for GA			
SGA (<10^th^ percentile)	-2.13 (-7.09, 2.83)	-1.28 (-6.48, 3.92)	-1.34 (-6.59, 3.91)
Not SGA (≥10^th^ percentile)	Ref	Ref	Ref

Model 0 = unadjusted; Model 1 = adjusted for maternal age, parity, wealth index, and maternal education; Model 3 = model 2 + breastmilk true protein at each timepoint. Abbreviations: MUAC = mid upper arm circumference; BMI = body mass index; GA = gestational age; BW = birthweight; SGA small-for-gestational age. * = p < 0.05; ^ = p < 0.10.

## 4. Discussion

Within this rural Bangladeshi cohort, we found that human milk lactoferrin concentrations increased over time. We also found that higher maternal early pregnancy MUAC and lower gestational age at birth were associated with higher lactoferrin.

Within our cohort, median human milk lactoferrin concentration was 13.56 mg/dL at T1 and 16.79 mg/dL at T2. These levels are lower than those previously reported in the literature in both developed and developing countries [7]. However, this difference in absolute concentration can be attributed to two key methodologic differences. We measured lactoferrin concentrations in whole milk [28], not skim milk as many previous reports have done, given that whole milk reflects infants’ intake more fully than skim milk, and lactoferrin is embedded within the milk fat globule membrane [30]. We also utilized electrochemiluminescence multiplex immunoassay technology using the mesoscale discovery platform to measure lactoferrin concentrations, as opposed to ELISA, SDS-PAGE, radial immunodiffusion, and immunoelectrophoresis which were used in previous studies [7,8].

Our finding that human milk lactoferrin concentration increased slightly over the course of lactation is consistent with Czosnykowska-Łukacka et al who demonstrated a trend towards a positive correlation between lactoferrin concentration and month of lactation from the first to the 48^th^ month [6]. However, many other studies have found that concentrations of lactoferrin decrease during early lactation and remain relatively stable after 1-month postpartum [7,8,31–38]. In studies conducted in low- and middle-income countries, Lonnerdal et al showed that human milk lactoferrin concentration was lower in mothers with postpartum infections [39] and other studies have shown that human milk lactoferrin concentration is higher in women with mastitis [40,41]. Furthermore, Rai et al reported in a global systematic review that lactoferrin concentration in term milk from Asia was higher from 2 weeks to >12 months compared to other regions [8], indicating there may be additional factors, such as infection or inflammation, that may be driving a preservation, or even slight increase, of lactoferrin concentration over time in this population. We did not collect data on postpartum infections or mastitis and could not test this hypothesis.

In this cohort, higher gestational age at delivery was associated with lower human milk lactoferrin concentration at T1. Furthermore, there was a trend towards preterm milk having higher lactoferrin concentration at T1 compared to term milk. Although our study population was mostly term infants (94% term), this finding aligns with previous research that found that lactoferrin levels are higher in preterm compared to term human milk [7,37,42]. Albenzio et al found that gestational age was negatively correlated with lactoferrin concentrations in colostrum, but not mature milk [37]. To our knowledge, no other studies have investigated the associations between continuous gestational age and lactoferrin concentrations in a population-based sample.

We found that higher maternal MUAC at baseline was associated with higher lactoferrin concentrations at T2. There was also a trend towards higher BMI at baseline, and overweight or obesity at five months, being associated with higher lactoferrin concentrations at T2. This finding is consistent with a study by Liang et al. that found that in a cohort of Chinese women that those with normal weight, overweight, and obesity had higher lactoferrin levels in human milk [43]. Our findings are also consistent with those of Rahfiludin et al., who reported in a cohort of women in Indonesia that higher MUAC was positively associated with human milk lactoferrin concentration, however they found no correlation between maternal BMI during lactation and lactoferrin concentration [44]. It is possible that maternal inflammation could be driving the increase in lactoferrin in human milk of mothers with overweight and obesity as higher inflammation has previously been associated with higher lactoferrin in infants less than five months of age [45]. However, our population had relatively low BMI and MUAC, with a median BMI at baseline of 19.4 kg/m^2^, and our findings need to be interpreted within that context. Given the distribution of BMI, our findings suggest that decreasing the burden of undernutrition might increase human milk lactoferrin concentrations in settings where maternal undernutrition is common, but findings may not generalize to well-nourished, high BMI populations. Our findings support the concept that improving maternal nutritional status prior to pregnancy and during the postpartum period should be a public health priority in undernourished populations for improving human milk composition.

Despite lactoferrin being an iron-binding protein, we found no associations between maternal hemoglobin and human milk lactoferrin concentrations at either timepoint. This finding is consistent with previous research in low and middle income countries that found no difference in human milk lactoferrin concentration between anemic and non-anemic mothers [38,45,46] and no correlation between maternal hemoglobin and human milk lactoferrin [44]. Zavaleta et al further reported that iron treatment did not affect human milk lactoferrin concentrations among the anemic women [46].

Our study had several strengths, including the availability of two human milk samples allowing for longitudinal assessment of lactoferrin and its predictors. Our study was also unique in that the population had a high rate of undernutrition with 33% of participants being underweight and 55% having low MUAC at baseline. We had high quality gestational age dating by early ultrasound and a thoroughly characterized pregnancy cohort allowing for adjustment for important confounders.

Limitations of our study included its small sample size, which limits our ability to detect small or moderate-sized associations that may be clinically relevant. We also did not collect data on maternal infections in the postpartum period which limited our ability to test our hypothesis that differing rates of postpartum infections may be driving the increase in human milk lactoferrin that we observed over time. Due to the high rates of direct breastfeeding in this population, we were unable to collect a human milk sample from a complete expression and instead used a mid-feed expression, however it has been shown that lactoferrin concentration does not differ between foremilk and hindmilk [5]. Another limitation is that we made multiple comparisons, however we did not adjust for multiple comparisons due to the exploratory nature and because we did not intend to reject a universal null hypothesis. Our study also only had a small number of preterm infants.

In this rural Bangladeshi cohort, we found that human milk lactoferrin increased over the course of lactation and that lower gestational age at delivery was associated with higher lactoferrin concentration. Further, better maternal nutritional status was associated with higher human milk lactoferrin. Future research should examine the impact of these findings on child health outcomes and determine the utility of maternal nutritional interventions on increasing human milk lactoferrin within low- and middle-income country settings.

## Supporting information

S1 Table Population characteristics of mother-infant dyads by tertile of human milk lactoferrin concentration at five months. Data presented are median (IQR) or n (%). ^a^Kruskal Wallis test for continuous variables and Pearson’s Chi^2^ or Fisher’s exact (when a cell had n<5) test for categorical variables. *p<0.05. Abbreviations: BMI=body mass index; MUAC=mid upper arm circumference; Hgb=hemoglobin; PHQ-9=Patient Health Questionnaire-9; GA=gestational age; SGA=small for gestational age; IQR=interquartile range.
(XLSX)

S1 Checklist Inclusivity in global research. (DOCX)

S1 Data Determinants of HM Lactoferrin - Manuscript Data. (XLS)
